# Resolution of Treatment-Refractory Papulopustular Rosacea With Combined Light Source Photodynamic Therapy Using Pre-incubation Nonablative Fractionated Laser Resurfacing

**DOI:** 10.7759/cureus.74502

**Published:** 2024-11-26

**Authors:** Merry A Mathew, Kritin K Verma, Daniel Friedmann, Jessica Tran, Vineet Mishra

**Affiliations:** 1 School of Medicine, Texas Tech University Health Sciences Center, Lubbock, USA; 2 Dermatology, Westlake Dermatology & Cosmetic Surgery, Austin, USA; 3 Dermatology, McGovern Medical School, Houston, USA; 4 Dermatology, University of California, San Diego, USA

**Keywords:** 5-aminolevulinic acid, blue light, demodex folliculorum, intense pulsed light, protoporphyrin ix, pulsed dye laser, red light

## Abstract

Papulopustular rosacea is an inflammatory subtype of rosacea that can significantly impair patients’ quality of life. Available treatment options range from anti-inflammatory topical and oral medications to laser and light therapies. Photodynamic therapy with aminolevulinic acid (ALA-PDT) has emerged as a more recent treatment option for papulopustular rosacea. ALA-PDT can significantly reduce the inflammatory component of rosacea, and pre-incubation nonablative fractionated laser resurfacing (NAFR) may enhance transcutaneous delivery of topical ALA. We present a case in which treatment-refractory papulopustular rosacea of 10-year duration was resolved with ALA-PDT using pre-incubation NAFR. This case highlights a novel, alternative approach to rosacea treatment that may be useful for patients recalcitrant to more conventional therapies.

## Introduction

Papulopustular rosacea is an inflammatory form of rosacea marked by recurring or persistent papules and pustules on the central face, accompanied by background erythema. This subtype frequently impacts middle-aged individuals and can significantly affect their quality of life. Sun avoidance, camouflaging with cosmetics and concealers, and anti-inflammatory topical medications (e.g., metronidazole, azelaic acid, ivermectin, and sodium sulfacetamide and sulfur) and second-generation tetracycline oral antibiotics (e.g., doxycycline and minocycline) have been the mainstays of therapy [1]. Light-based treatments like pulsed dye laser (PDL) and intense pulsed light (IPL) are also effective in reducing erythema, inflammatory lesions, and telangiectasias. Photodynamic therapy with aminolevulinic acid (ALA-PDT) is an emerging treatment method that may produce significantly longer-term improvements in the signs and symptoms of rosacea [1]. Moreover, pre-incubation nonablative fractionated laser resurfacing (NAFR) has been shown to enhance the transcutaneous delivery of topical ALA, which could further optimize results [2].

This article was previously presented as a meeting abstract at the 2017 ASLMS Annual Conference on April 8, 2017.

## Case presentation

A 52-year-old, type II Fitzpatrick skin type, Caucasian female patient presented with moderate papulopustular rosacea and background erythema, primarily affecting her nose, glabella, and mid-face. The signs and symptoms of rosacea began 10 years earlier, with no significant lesion-free periods during that time. She was unable to tolerate topical metronidazole and azelaic acid due to excessive irritation and dryness, as well as doxycycline and minocycline due to diarrhea. The patient also failed to respond to topical clindamycin. Combination topical ivermectin cream and four monthly sessions of IPL using a 560-nm cutoff filter with double-pulse durations of 3-6 ms, a delay of 15 ms, and fluences of 18-20 J/cm^2^ produced only marginal, short-term improvement and poor patient satisfaction.

Given the refractory nature of her rosacea, the patient was treated with combined light source ALA-PDT using pre-incubation NAFR. Two sessions were performed three months apart. As part of the informed consent process, the off-label use of this treatment for rosacea was thoroughly explained before therapy began. During each session, the patient’s face was cleansed with 99.5% acetone, and topical anesthesia (23% lidocaine/7% tetracaine) ointment was applied for 30-60 minutes. NAFR (ResurFX, Lumenis Ltd., Yokneam, Israel) was first performed on the affected areas of the face with a fluence of 50-60 mJ and a density of 200-250 spots/cm^2^ using integrated contact cooling. ALA was then incubated unoccluded for one hour in a dimly lit room. PDT was started with 417-nm blue light-emitting diode light (BLU-U, DUSA Pharmaceuticals Inc., Wilmington, MA, United States) for 16 minutes and 40 seconds with a fluence dose of 10 J/cm^2^. Field treatment with PDL (VBeam Perfecta, Candela Corp., Wayland, MA, United States) was then performed using a 10-mm spot size, 40-ms pulse duration, and 7 J/cm^2^ fluence. The treatment concluded with 15 minutes of daylight exposure instead of a red light device [3]. Sunscreen containing titanium dioxide or zinc oxide was applied immediately after treatment. The patient was instructed to avoid direct sunlight for up to 48 hours.

**Figure 1:**
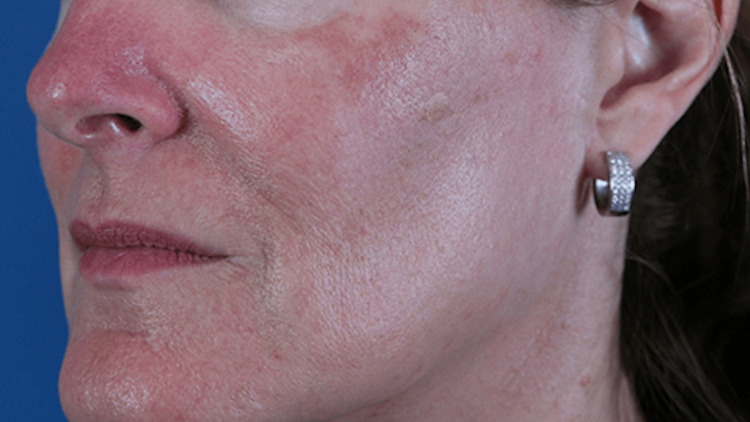
Erythema, post-inflammatory hyperpigmentation, and slight residual crusting at one month following the first treatment session. Adverse events were resolved in three months.

**Figure 2:**
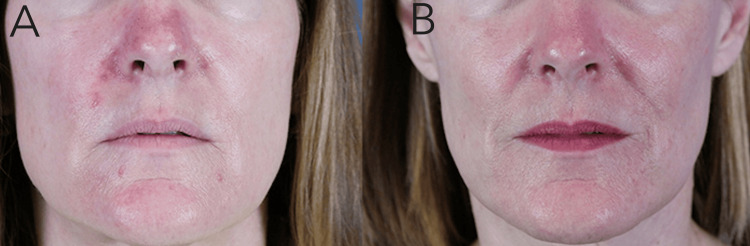
(A) Before and (B) three months after two sessions of photodynamic therapy with pre-incubation nonablative fractionated laser resurfacing.

Adverse events included transient erythema, mild-to-moderate crusting, and post-inflammatory hyperpigmentation (Figure 1). All adverse events were resolved without the need for additional treatment. One month following the second session, the patient reported complete resolution of her inflammatory papules and pustules and near-resolution of her background erythema (Figure 2). Results have been maintained at two years posttreatment.

## Discussion

ALA-PDT shows promise as a treatment option for papulopustular rosacea, potentially eliminating the need for prolonged use of medications and offering an effective option for patients unresponsive to standard therapies. ALA is preferentially absorbed by the sebaceous glands and metabolized into highly photoactive protoporphyrin IX (PpIX). Cutaneous irradiation with a light source emitting a wavelength in the absorption spectrum of PpIX (410 nm, 500-630 nm) triggers the production of singlet oxygen and reactive oxygen species (ROS). These cytotoxic molecules may injure hyperplastic and inflamed pilosebaceous units and reduce their population of *Demodex folliculorum* [4].

The use of multiple sequential laser and light sources (e.g., 417-nm blue light, 635-nm red light, PDL, and IPL) may potentially maximize porphyrin photobleaching without increasing adverse events [5,6]. The superficial anti-inflammatory and epidermal turnover effects of blue light may act synergistically with the antimicrobial action of deeply penetrating red light. Adding PDL (585-595 nm) or IPL (560-1200 nm) targets oxyhemoglobin’s absorption peak at 577 nm, enabling targeted photothermolysis of erythema and discrete telangiectasias.

A patient who underwent six sessions of ALA-PDT, as reported by Katz and Patel [1], demonstrated significant improvement after one month. This patient had 15-minute incubations followed by 15-minute ultrasound treatments to purportedly enhance topical delivery of ALA. In two other prospective studies evaluating its efficacy for rosacea, ALA-PDT demonstrated significant improvements in both objective measures and patient-reported outcomes. One study showed that after three treatments, 64.71% achieved at least 50% improvement, with reductions in erythema index and high patient satisfaction scores [7]. Another study confirmed clinical improvement in all patients after four sessions, with inflammatory lesions disappearing completely by week 24 and no relapses in subjective symptoms such as flushing or burning. While side effects like pain, erythema, and swelling were noted, they were transient and well-tolerated [8]. A systematic review of nine studies evaluating methyl aminolevulinate (MAL)-PDT and ALA-PDT for rosacea suggests that while PDT shows promising safety and effectiveness, more rigorous trials are needed to confirm these results [9].

Although the gold standard for enhanced delivery of topical products across the skin barrier has been ablative AFR, this can also be achieved with NAFR. These devices produce columnar microthermal treatment zones of dermal coagulation necrosis based on selective photothermolysis of water molecules, sparing the overlying epidermis and limiting skin barrier disruption compared to their ablative counterparts. A study of 10 subjects by Lim et al. [2] demonstrated that the use of a 1550-nm nonablative fractional erbium-glass laser pre-incubation led to significantly greater ALA penetration and porphyrin fluorescence compared to areas not pretreated with NAFR. Increased laser fluence (20 mJ vs. 50 mJ) and ALA incubation time (30 vs. 60 vs. 180 minutes) were positively correlated with ALA absorption.

## Conclusions

We report successful long-term control of papulopustular rosacea with two sessions of combined light source ALA-PDT using pre-incubation NAFR in a patient who had failed prior pharmacologic, light, and laser therapies. The patient experienced complete resolution of inflammatory papules and pustules, as well as near-complete resolution of background erythema, with no significant relapses or need for further treatment for two years posttreatment. Pre-incubation NAFR may play an important role in enhancing ALA penetration while minimizing adverse events associated with AFR. Further randomized controlled trials with a larger sample size and extended follow-up periods are needed to confirm the efficacy and safety of this combined treatment approach. This case highlights the potential of ALA-PDT with pre-incubation NAFR as an effective and durable therapeutic option for rosacea patients who fail to respond to conventional pharmacologic treatments and light or laser therapies.
